# Incidence of and risk factors for postoperative delirium in older adult patients undergoing noncardiac surgery: a prospective study

**DOI:** 10.1186/s12877-020-1449-8

**Published:** 2020-02-03

**Authors:** Arissara Iamaroon, Titima Wongviriyawong, Patumporn Sura-arunsumrit, Nattikan Wiwatnodom, Nichakarn Rewuri, Onuma Chaiwat

**Affiliations:** 1 Department of Anesthesiology, Faculty of Medicine Siriraj Hospital, Mahidol University, Bangkok, 10700 Thailand; 2 Division of Geriatric Medicine, Department of Medicine, Faculty of Medicine Siriraj Hospital, Mahidol University, Bangkok, 10700 Thailand; 3 Integrated Perioperative Geriatric Excellent Research Center, Faculty of Medicine, Siriraj Hospital, Mahidol University, Bangkok, 10700 Thailand

**Keywords:** Collaborative approach, Incidence, Risk factors, Postoperative delirium, Older adult patients, Noncardiac surgery

## Abstract

**Background:**

To identify the incidence of, risk factors for, and outcomes associated with postoperative delirium (POD) in older adult patients who underwent noncardiac surgery.

**Methods:**

This prospective study recruited patients aged ≥ 60 years who were scheduled to undergo noncardiac surgery at Siriraj Hospital (Bangkok, Thailand). Functional and cognitive statuses were assessed preoperatively using Barthel Index (BI) and the modified Informant Questionnaire on Cognitive Decline in the Elderly, respectively. POD was diagnosed based on the Diagnostic and Statistical Manual of Mental Disorders Fifth Edition criteria. Incidence of POD was reported. Univariate and multivariate analyses were used to identify risk factors for POD.

**Results:**

Of the 249 included patients, 29 (11.6%) developed POD. Most patients (61.3%) developed delirium on postoperative day 1. Univariate analysis showed age ≥ 75 years, BI score ≤ 70, pre-existing dementia, preoperative use of opioid or benzodiazepine, preoperative infection, and hematocrit < 30% to be significantly associated with POD. Multivariate logistic analysis revealed pre-existing dementia (adjusted risk ratio [RR]: 3.95, 95% confidence interval [CI]: 1.91–8.17; *p* < 0.001) and age ≥ 75 years (adjusted RR: 2.54, 95% CI: 1.11–5.80; *p* = 0.027) to be independent risk factors for POD. Median length of hospital stay was 10 (range: 3–36) days for patients with POD versus 6 (range: 2–76) days for those without delirium (*p <* 0.001).

**Conclusions:**

POD remains a common surgical complication, with an incidence of 11.6%. Patients with pre-existing dementia and age ≥ 75 years are the most vulnerable high-risk group. A *multidisciplinary team consisting of anesthesiologists and geriatricians should implement perioperative care to prevent and manage* POD.

## Background

Delirium is a common postoperative complication that occurs in 5 to 52% of older adult patients after noncardiac surgery [1, 2]. Delirium is characterized by disturbance in attention, awareness, and cognition that develops acutely and fluctuates frequently throughout the course of the condition [3]. POD adversely impacts patient quality of life, *and increases the burden on the patient’s family*. Delirium has been associated with adverse outcomes, such as functional decline [4, 5], dementia or cognitive impairment [6, 7], increased hospital length of stay [8, 9], increased mortality [7–9], institutionalization [7, 8], and increased healthcare costs [10].

As the population of older adults increases, so too with the number of older adult *patients that present for anesthesia and surgery.* The *development* of delirium following surgery has some significant potential effects on patient outcomes; however, POD is often underdiagnosed. Some studies reported that more than 50% of patients with delirium were undiagnosed by medical teams [11–13]. Moreover, it is sometimes difficult to differentiate delirium, especially hypoactive delirium, from the residual effects of anesthesia during the early postoperative period [14]. Delirium has multifactorial causes and complex pathophysiological mechanisms. Clinical studies of incidence and risk factors associated with POD may provide additional useful clues to the optimal perioperative care of older adult surgical patients at risk for delirium. Risk identification may also help clinicians provide patient-specific management during the perioperative period.

The gold standard diagnostic criteria for delirium is the Diagnostic and Statistical Manual of Mental Disorders Fifth Edition (DSM-5) from the American Psychiatric Association [3]. Definitive delirium diagnosis should be performed by a trained and experienced physician, such as a geriatrician or psychiatrist. A standardized diagnostic tool used by a trained and experienced physician may help to maximize the detection of POD. Traditionally, geriatric consultation is usually activated once the patient develops delirium after an operation. Proactive geriatric consultation together with the careful anesthesia techniques for surgical patients who have risk for delirium may reduce the incidence of POD and its associated adverse outcomes during the perioperative period. A collaborative approach between geriatricians and anesthesiologists may also improve the quality of patient care and patient outcomes. Before a program with these objectives can be developed and implemented, the scope of the problem and factors that significantly associate with POD must be identified.

Accordingly, the aim of this study was to enlist anesthesiologists and geriatricians to collaboratively investigate the incidence of, risk factors for, and outcomes associated with POD among older adult patients who underwent noncardiac surgery.

## Methods

After receiving Siriraj Institutional Review Board (COA no. Si 718/2015) approval, a prospective cohort study was conducted at a large university-based national tertiary referral center during the March 2017 to December 2017 study period. Patients aged 60 years or older who were scheduled to undergo noncardiac surgery were eligible for inclusion. Patients were excluded if they refused to participate in the study or required postoperative intensive care unit admission. The protocol for this study followed all of the guidelines outlined in the Declaration of Helsinki and all of its later amendments. Written informed consent was obtained from all study participants.

Preoperatively, all patients were assessed for functional and cognitive status by the clinical researcher or a trained research assistant. Functional status was assessed using the Barthel Index of Activities of Daily Living [15]. The Barthel Index (BI) consists of 10 items that assess self-care abilities, including feeding, grooming, bathing, dressing, toilet use, bowel and bladder control, mobility, stair climbing, and transferring from bed to chair. Scoring ranges from 0 (totally dependent) to 100 (fully independent). The rating was classified as ≤ 70 or > 70. The information was obtained from an observation of patient performance or a caregiver interview.

Cognitive status was measured using the modified Informant Questionnaire on Cognitive Decline in the Elderly (IQCODE), which is based on information elicited from a close relative or caregiver of each patient. The modified IQCODE for detection of dementia in Thai older adults (90% sensitivity, 95% specificity, 94% positive predictive value, and 90% negative predictive value) consists of 32 items designed to assess cognitive changes over the previous 10 years [16]. Scoring ranges from 1 (much improved) to 5 (much worse), and total scores were divided by the number of items (32) to give an average score of 1–5, with an optimal cutoff score of 3.42. Accordingly, patients with a modified IQCODE score greater than or equal to 3.42 were considered to have some degree of dementia [16]. Patients with a pre-existing diagnosis of dementia were also obviously similarly classified.

POD was diagnosed by one of three geriatricians based on the DSM-5 criteria [3]. All three geriatricians have had experience in caring for older adult patients with delirium and dementia for an experience duration ranging from 5 to 15 years. To enhance agreement among geriatricians relative to delirium diagnosis, a DSM-5 interrater reliability among these three geriatricians was measured, and a level of agreement ranging from 90 to 100% was obtained. *Daily* patient assessment for POD was performed during the daytime for 7 consecutive days after surgery. The occurrence of POD and *delirium* onset time was documented.

The surgical procedures and anesthetic techniques were carried out with no specific intervention from the research team. Patient demographic data, comorbidities, type and duration of surgery, anesthesia technique, intraoperative hypotension, intraoperative hypoxemia, and length of hospital stay were recorded. Intraoperative hypotension was defined as either systolic blood pressure less than 90 mmHg for more than 5 min or the use of vasopressor to treat hypotension [17]. Intraoperative hypoxemia was defined as oxygen saturation, measured by pulse oximeter, of below 90% for any duration [18].

Medications used within 3 months before surgery were also recorded, including opioids, benzodiazepines, statins, anticonvulsants, and psychotropic drugs (antipsychotics, antidepressants, and antianxiety drugs were grouped together as psychotropic drugs). Preoperative laboratory values, including hematocrit (Hct), sodium, and ratio of blood urea nitrogen to creatinine (BUN/Cr ratio), were measured and recorded. Maximum pain scores on the first postoperative day were determined using a *numeric* rating *scale* (NRS) on a 10-point scale. Pain intensity was classified as *mild* (NRS = 1–3), *moderate* (NRS = 4–7), or *severe* (NRS = 8–10) pain.

### Statistical analysis

The sample size was estimated based on multiple logistic regression analysis [19]. Based on literature review, the risk factors for POD were approximately 10 variables [2] and the number of patients with delirium should be 5 to 10 times of risk factors. From the previous study [8], the incidence of POD was 44% among patients undergoing noncardiac surgery. The sample size was calculated and a minimum sample size of 227 patients was required. To compensate for a possible 10% dropout rate for any reason, a minimum total study population of 250 patients was required.

All statistical analyses were performed using PASW Statistics version 18.0 (SPSS, Inc., Chicago, IL, USA) and MedCalc Statistic Software version 17.6 (MedCalc Software BVBA, Ostend, Belgium). Continuous data are presented as mean ± standard deviation (SD) for normally distributed data, and as median and interquartile range for non-normally distributed data. Categorical data are presented as frequency and percentage. All variables in patients without POD versus those with POD were analyzed by univariate logistic regression analysis using chi-square test, Fisher’s exact test, independent *t*-test, or Mann-Whitney U test, as appropriate. Seven risk factors with univariable *p*-value less than 0.05 including preexisting dementia, age ≥ 75 years, hematocrit < 30%, preoperative opioid use, preoperative benzodiazepine use, preoperative infection, and Barthel index score ≤ 70 were entered into multiple regression model. Risk ratios and adjusted risk ratios with their respective 95% confidence intervals were reported. A *p-*value less than 0.05 was considered to be statistically significant for all tests. Receiver operating characteristic (ROC) curve analysis was performed to identify the optimal cutoff age for developing delirium. The results of that analysis are reported as Youden’s index, sensitivity, specificity, positive predictive value, negative predictive value, positive likelihood ratio, negative likelihood ratio, and area under curve.

## Results

**Fig. 1:**
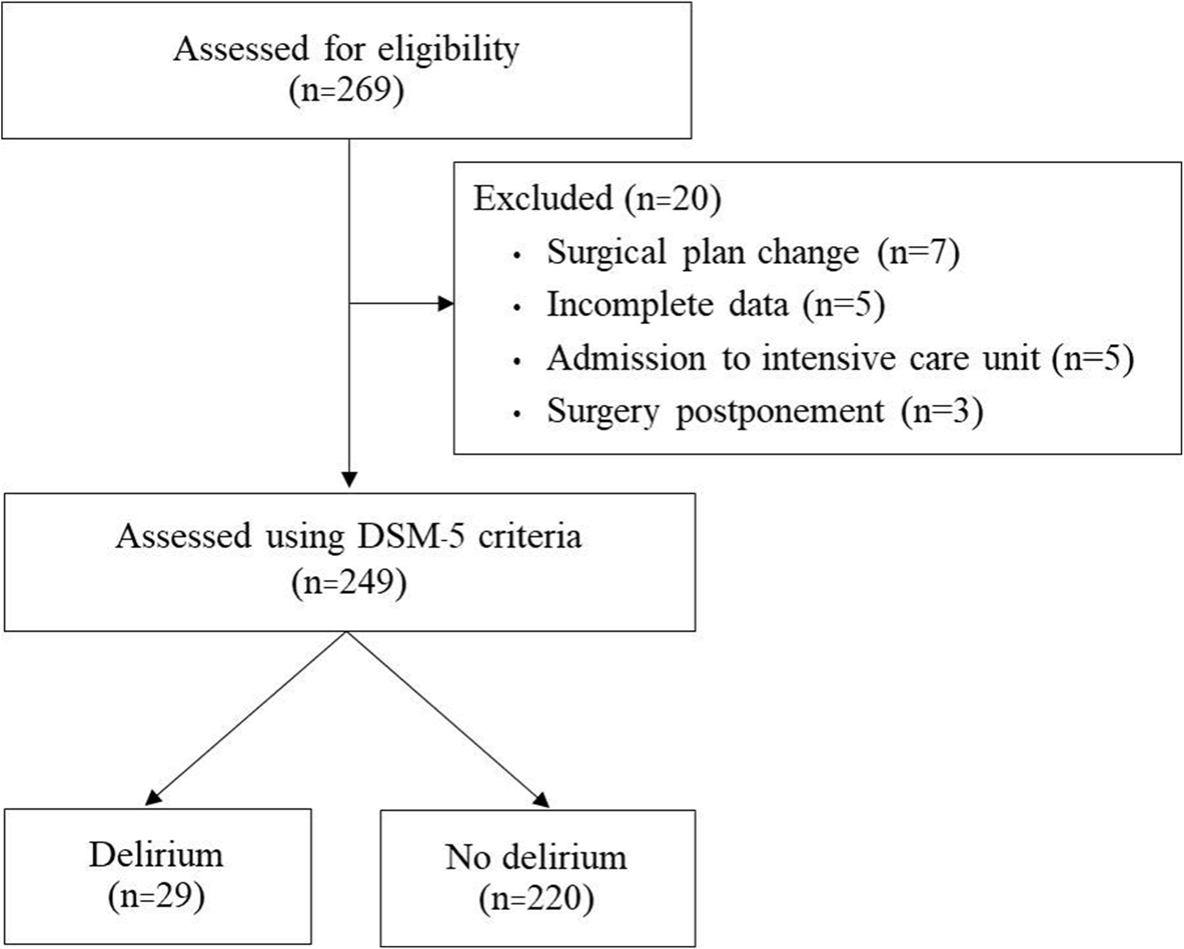
Flow diagram of patient enrollment

During the study period, a total of 269 patients were assessed for eligibility. Of those, 20 patients were excluded for the reasons showed in Fig. 1. The remaining 249 patients were included and completed the study. The demographic and clinical data of patients are summarized in Table 1. Perioperative data is described in Table 2.

**Table 1:** Demographic and clinical data of patients without and with delirium

Variables	Total (*n* = 249)	No delirium (*n* = 220; 88.4%)	Delirium (*n* = 29; 11.6%)	*p*-value
Age (years), mean ± SD	75.1 ± 7.9	74.4 ± 6.9	80.7 ± 6.6	< 0.001
Age ≥ 75 years, n (%)	125 (50.2)	102 (46.4)	23 (79.3)	0.001
Female gender, n (%)	154 (61.8)	137 (62.3)	17 (58.6)	0.839
ASA physical status, n (%)				0.545
II	152 (61.0)	136 (61.8)	16 (52.5)	
III	97 (39.0)	84 (38.2)	13 (44.8)	
Barthel Index scores ≤ 70, n (%)	51 (20.5)	38 (17.3)	13 (44.8)	0.001
Alcohol ≥ 2 drinks daily, n (%)	83 (33.3)	73 (33.2)	10 (34.5)	1.000
Smoking ≥ 30 pack year, n (%)	68 (27.3)	61 (27.7)	7 (24.1)	0.826
Comorbidities, n (%)				
Diabetes mellitus	84 (33.7)	78 (35.5)	6 (20.7)	0.144
Hypertension	184 (73.9)	165 (75.0)	19 (65.5)	0.368
Heart disease	43 (17.3)	36 (16.4)	7 (24.1)	0.433
Stroke or TIA	13 (5.2)	12 (5.5)	1 (3.4)	1.000
ESRD or CKD	54 (21.7)	48 (21.8)	6 (20.7)	1.000
Cirrhosis	5 (2.0)	5 (2.3)	0 (0.0)	1.000
Cancer	55 (22.1)	45 (20.5)	10 (34.5)	0.098
Dementia	42 (16.9)	26 (11.8)	16 (55.2)	< 0.001
Preoperative infection	24 (9.6)	18 (8.2)	6 (20.7)	0.044
Preoperative medications, n (%)				
Opioids	36 (14.5)	25 (11.4)	11 (37.9)	0.001
Benzodiazepines	37 (14.9)	28 (12.7)	9 (31.0)	0.022
Psychotropics	14 (5.6)	11 (5.0)	3 (10.33)	0.214
Anticonvulsants	19 (7.6)	14 (6.4)	5 (16.1)	0.054
Statins	136 (54.6)	124 (56.4)	12 (41.4)	0.165

A *p*-value < 0.05 indicates statistical significance

*Abbreviations*: *SD* standard deviation, *ASA* American Society of Anesthesiologists, *TIA* transient ischemic attack, *ESRD* end-stage renal disease, *CKD* chronic kidney disease

**Table 2:** Perioperative data of patients without and with delirium

Variables	Total (*n* = 249)	No delirium (*n* = 220)	Delirium (*n* = 29)	*p*-value
*Type of surgery*, n (%)				0.606
Vascular	10 (4.0)	8 (3.6)	2 (6.9)	
Urological	26 (10.4)	24 (10.9)	2 (6.9)	
General	94 (37.8)	85 (38.6)	9 (31.0)	
Orthopedic	119 (47.8)	103 (46.8)	16 (55.2)	
Urgent surgery, n (%)	38 (15.3)	30 (13.6)	8 (27.6)	0.058
Operative time (min), mean ± SD	115.3 ± 70.0	115.3 ± 68.9	115.3 ± 79.0	0.997
Type of anesthesia, n (%)				0.659
General	142 (57.0)	126 (57.3)	16 (55.2)	
Regional	85 (34.1)	76 (34.5)	9 (31.0)	
Combined	22 (8.8)	18 (8.2)	4 (13.8)	
Intraoperative benzodiazepines, n (%)	55 (22.1)	50 (22.7)	5 (17.2)	0.637
Intraoperative anticholinergics, n (%)	143 (57.4)	128 (58.2)	15 (51.7)	0.552
Intraoperative hypotension, n (%)	127 (51.0)	108 (49.1)	19 (65.5)	0.115
Intraoperative hypoxemia, n (%)	0 (0)	0 (0)	0 (0)	–
Intraoperative blood loss (mL), median (IQR)	50 (20–200)	50 (20–175)	100 (30–250)	0.143
Intraoperative blood transfusion, n (%)	19 (7.6)	15 (6.8)	4 (13.8)	0.252
Postoperative pain intensity^a^, n (%)				0.142
No pain	32 (12.9)	28 (12.7)	4 (13.8)	
Mild pain (NRS = 1–3)	96 (38.6)	88 (40.0)	8 (27.6)	
Moderate pain (NRS = 4–6)	86 (34.5)	77 (35.0)	9 (31.0)	
Severe pain (NRS = 7–10)	35 (14.1)	27 (12.3)	8 (27.6)	
Abnormal laboratory values, n (%)				
Preoperative hematocrit < 30%	35 (14.1)	27 (12.3)	8 (27.6)	0.042
Preoperative sodium <135 or > 145 mEq/L	24 (9.6)	20 (9.1)	4 (13.8)	0.498
Preoperative BUN/Cr ratio > 20	62 (24.9)	53 (24.1)	9 (31.0)	0.493
Length of stay (days), median (IQR)	7 (2–76)	6 (2–76)	10 (3–36)	< 0.001
Length of stay > 7 days, n (%)	103 (41.4)	78 (35.5)	25 (86.2)	< 0.001

A *p*-value < 0.05 indicates statistical significance

*Abbreviations*: *SD* standard deviation, *IQR* interquartile range, *BUN/Cr* blood urea nitrogen/creatinine

^a^Maximum pain score using numeric rating scale (NRS) was measured on the first postoperative day

**Fig. 2:**
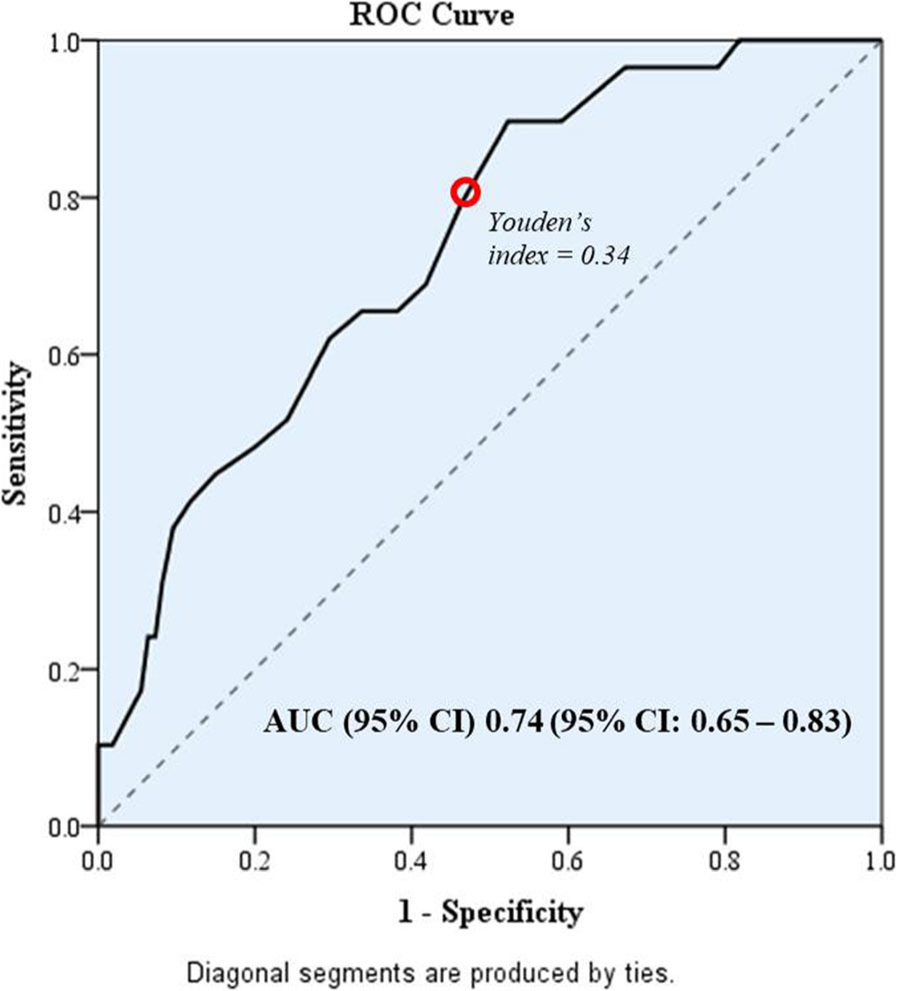
Receiver operating characteristic (ROC) curve of the age classification of delirious patients

Using DSM-5 criteria, 29 of 249 patients developed delirium, for an incidence of 11.6%. The majority of patients (61.3%) developed delirium on postoperative day 1, followed by 16.1% on day 2, 12.9% on day 3, 6.5% on day 5, and 3.2% on day 6. As shown in Table 1, patients with delirium were significantly older (*p* < 0.001), had a greater prevalence of pre-existing dementia (*p* < 0.001), had a lower Barthel Index score (*p* = 0.001), and had a higher rate of preoperative opioid (*p* = 0.001) or benzodiazepine (*p* = 0.022) relative to those patients without delirium. In addition, among patients with delirium versus those without delirium, hematocrit less than 30% (27.6% vs. 12.3%, respectively; *p* = 0.042) and preoperative infection (20.7% vs. 8.2%, respectively; *p* = 0.044) were significantly associated with the development of delirium. Median length of hospital stay was 10 (range: 3–36) days for patients with delirium versus 6 (range: 2–76) days for those without delirium (*p <* 0.001). In multivariate analysis, only pre-existing dementia (adjusted risk ratio [RR]: 3.95, 95% confidence interval [CI]: 1.91–8.17; *p* < 0.001) and age ≥ 75 years (adjusted RR: 2.54, 95% CI: 1.11–5.80; *p* = 0.027) remained significantly associated with POD in patients undergoing noncardiac surgery (Table 3). The ROC curve, with its area under curve of 0.74 (95% CI: 0.65–0.83), is shown in Fig. 2. The optimal cutoff age for developing delirium was age ≥ 75 years. The sensitivity, specificity, positive predictive value, negative predictive value and Youden’s index for optimal cutoff age was 79.3, 53.6%, 18.4, 95.2, and 0.34, respectively (Table 4).

**Table 3:** Univariate and multivariate analysis for variables significantly associated with postoperative delirium

Variables	n	Delirium (n = 29)	Crude RR (95% CI)	*p*-value	Adjusted RR (95% CI)	*p*-value
Pre-existing dementia, n (%)						
No	207	13 (6.3)	1		1	
Yes	42	16 (38.1)	6.07 (3.16–11.65)	< 0.001	3.95 (1.91–8.17)	< 0.001
Age ≥ 75 years, n (%)						
No	124	6 (4.8)	1		1	
Yes	125	23 (18.4)	3.80 (1.60–9.03)	0.001	2.54 (1.11–5.80)	0.027
Preoperative hematocrit <30%, n (%)						
No	214	21 (9.8)	1		1	
Yes	35	8 (22.9)	2.33 (1.12–4.84)	0.042	1.53 (0.79–2.96)	0.204
Preoperative opioids, n (%)						
No	213	18 (8.5)	1		1	
Yes	36	11 (30.6)	3.62 (1.87–7.01)	0.001	1.80 (0.83–3.91)	0.139
Preoperative benzodiazepines, n (%)						
No	212	20 (9.4)	1		1	
Yes	37	9 (24.3)	2.58 (1.27–5.22)	0.022	1.41 (0.66–3.01)	0.370
Preoperative infection, n (%)						
No	225	23 (10.2)	1		1	
Yes	24	6 (25.0)	2.45 (1.11–5.41)	0.044	1.47 (0.68–3.19)	0.329
Barthel Index score ≤ 70, n (%)						
No	198	16 (8.1)	1		1	
Yes	51	13 (25.5)	3.15 (1.62–6.13)	0.001	1.14 (0.54–2.40)	0.734

A *p*-value < 0.05 indicates statistical significance

*Abbreviations*: *RR* risk ratio, *CI* confidence interval

**Table 4:** Receiver operating characteristic curve analysis of the optimal cutoff age for developing delirium

Cut point	Sensitivity (%)	Specificity (%)	PPV	NPV	LR+	LR-	Accuracy (95% CI)	Youden’s Index
≥ 65	100.00	9.09	12.66	100.0	1.10	0.00	19.68% (14.9–25.2)	0.09
≥ 70	96.55	21.82	14.00	97.96	1.23	0.16	30.52% (24.9–36.6)	0.18
≥ 75	79.31	53.64	18.40	95.16	1.71	0.39	56.63% (50.2–62.9)	0.34
≥ 80	51.72	75.91	22.06	92.27	2.15	0.64	73.09% (67.1–78.5)	0.28
≥ 85	31.03	91.82	33.33	90.99	3.79	0.75	84.74% (79.7–89.0)	0.23

*Abbreviations*: *PPV* positive predictive value, *NPV* negative predictive value, *LR+* positive likelihood ratio, *LR-* negative likelihood ratio, *AUC* area under the curve, *95% CI* 95% confidence interval

The optimal cutoff age was age ≥ 75 years with the best sensitivity and Youden's index

## Discussion

Based on DSM-5 criteria, this prospective cohort study found an 11.6% incidence of postoperative delirium among the older adult patients *admitted to the surgical ward after* noncardiac surgery. Pre-existing dementia and age 75 years or older were the independent risk factors for POD in these patients. In terms of outcome, patients who developed delirium had longer hospital stay than those without delirium. There was no in-hospital mortality in this study.

The 11.6% incidence of POD in the present study was similar to the 13.2% incidence reported in a previous study [9]. This is comparable with the 18.4% pooled incidence of POD reported by a systematic review of 41 studies [20]. In contrast, a high incidence rate of POD in the surgical intensive care unit has been reported to range from 24.4 to 44% [8, 21]. This reflects the fact that the reported incidence of POD varies from study to study depending on the patient population, timing of assessment, experience of the investigator, surgery type, and diagnostic tools to assess for delirium. Management goals for reducing the incidence and duration of POD should be included in the clinical guidelines or protocols.

Regarding onset time of POD, most patients in this study (61.3%) developed POD on postoperative day 1, whereas those in previous studies [22, 23] developed POD on postoperative day 2. However, a POD episode can occur any time during the entire postoperative period. According to the American Geriatrics Society Expert Panel, the clinical guideline for POD recommends that delirium assessment should be performed at least once daily in all patients at high risk for developing delirium [24, 25].

In the present study, pre-existing dementia was the strongest risk factor for POD, with an adjusted risk ratio of 3.95 (95% CI: 1.91–8.17). This finding is consistent with previous studies [6, 8, 26] that reported pre-existing dementia as a risk factor for the development of POD. A *recent* study of non-surgical patients also reported pre-existing dementia *to be* the main risk factor for delirium [27]. Delirium superimposed on dementia (DSD) is the term used for delirium that occurs in patients with pre-existing dementia [28]. The prevalence of DSD was reported to be as high as 22–89% in hospital and community populations [29], and 1.4–70% in institutionalized patients [30]. The occurrence of DSD can lead to poor delirium outcomes, including increased risk of mortality, institutionalization, and length of stay [31, 32]. Given the adverse outcomes of delirium, preventive strategies should be implemented to these patients. The delirium prevention strategies include reorientation, hydration, optimized oxygenation, infection control, early mobility, appropriate pain management, medication review, nutrition support, hearing and visual aids, and sleep hygiene [25].

Older age is also an important risk factor for delirium. The present study showed a significant association between POD and age 75 *years or older*, with an adjusted risk ratio of 2.54 (95% CI: 1.11–5.80). From previous studies, there were different age groups of patients (e.g., age ≥ 65, age ≥ 70, and age ≥ 75) that were identified as groups at high risk for delirium [2, 9, 25]. In the present study, ROC curve analysis revealed age ≥ 75 years to be the optimal cutoff age for developing delirium, with a sensitivity of 79.3%. This finding suggests that delirium screening might be performed in postoperative noncardiac patients that are aged 75 *years or older.* Although age is a fixed risk factor that cannot be modified, the delirium prevention and management strategies may be of benefit via their effects on intensity and duration of delirium.

Previous studies revealed that delirium may be preventable in 30–40% of hospitalized older adult patients [33, 34]. Identification of high-risk patients may be helpful in prevention of delirium. The present study demonstrated that patients with pre-existing dementia and age 75 years or older are the most vulnerable high-risk groups. These findings suggest the routine screening for delirium in these groups of patients. Proactive geriatric consultation focusing on these patients may decrease the risk of POD or reduce its severity. Importantly, the involved healthcare professionals should work together as a multidisciplinary team for successful management of POD.

Strengths of the present study include its prospective design, the fact that we used the DSM-5 for diagnosing delirium, and that delirium diagnosis was made by experienced geriatricians. Some limitations must also be mentioned. First, this study was conducted at a single institution. Therefore, our results may not be generalizable to other care settings. Second, the sample size may be too small to identify all significant differences and associations that relate to POD. Third, delirium assessment was only performed once daily, so the incidence of POD may have been underestimated. Finally, some relevant information was not obtained, such as prior postoperative delirium and intraoperative medication usage, and this could have had a confounding effect on our analysis and findings.

## Conclusions

This prospective study found an 11.6% incidence of postoperative delirium among patients undergoing noncardiac surgery. Pre-existing dementia and age 75 *years or older were found to be the independent risk factors for development of* POD. Collaborative approach to identify patients at risk for delirium and provide perioperative management strategies may help to prevent POD or reduce its severity. Further studies that focus on *multidisciplinary collaboration* are needed to improve delirium care.

## Data Availability

The datasets used and/or analyzed during the current study are available from the corresponding author on reasonable request. The datasets generated and/or analyzed during the present study are not publicly available due to internal institutional restrictions, but they are available from the corresponding author on reasonable request and with the permission of the institution where the data was generated.

## Abbreviations

95% CI: 95% confidence interval
ASA: American Society of Anesthesiologists
AUC: Area under the curve
BUN/Cr: Blood urea nitrogen/creatinine
CKD: Chronic kidney disease
DSD: Delirium superimposed on dementia
DSM-5: Diagnostic and Statistical Manual of Mental Disorders Fifth Edition
ESRD: End-stage renal disease
IQCODE: Informant Questionnaire on Cognitive Decline in the Elderly
LR-: Negative likelihood ratio
LR+: Positive likelihood ratio
NPV: Negative predictive value
POD: Postoperative delirium
PPV: Positive predictive value
ROC: Receiver operating characteristic
RR: Risk ratio
TIA: Transient ischemic attack
